# Validity of multiple human pose estimation tools for measuring knee impact angles in video-captured falls of older adults

**DOI:** 10.1371/journal.pone.0335108

**Published:** 2026-07-09

**Authors:** Reese Michaels, Justin Ehrlich, Yajun Mei, Jongsang Son, Stephen N. Robinovitch, Jacob J. Sosnoff, Yaejin Moon

**Affiliations:** 1 Department of Exercise Science, Syracuse University, Syracuse, New York, United States of America; 2 Department of Sport Analytics, Syracuse University, Syracuse, New York, United States of America; 3 Department of Biostatistics, New York University, School of Global Public Health, New York, New York, United States of America; 4 Department of Biomedical Engineering, New Jersey Institute of Technology, Newark, New Jersey, United States of America; 5 Department of Biomedical Physiology and Kinesiology, Simon Fraser University, Burnaby, Canada; 6 Department of Physical Therapy, Rehabilitation Science, & Athletic Training, University of Kansas Medical Center, Kansas, Kansas, United States of America; INAIL: Istituto Nazionale Assicurazione Contro gli Infortuni sul Lavoro, ITALY

## Abstract

Falls are a major cause of injury in older adults. Although bending the knees during a fall has been shown to reduce stress on the hip, knee motion during falls is not well understood because laboratory fall studies are limited by safety concerns and marker occlusion in motion capture systems. AI-based pose estimation may help overcome these challenges, but its accuracy in measuring joint angles during falls has not yet been validated. We evaluated three pose estimation models (OpenPose, VideoPose3D, WHAM) for analyzing knee kinematics in video-captured falls. A total of 121 videos of 13 older adults (64.0 ± 5.9 years) falling sideways, utilizing diverse fall strategies (knee block, stick-like, tuck-and-roll), in a lab setting were analyzed. Each model generated time series of knee angles from the videos, from which knee flexion angles at ground impact were calculated and compared to ground truth data from a motion capture system. Agreement with the ground truth was assessed using mean absolute error (MAE), mean absolute percentage error (MAPE), and bias, analyzed across viewing planes (sagittal vs. frontal) and leg sides (impact vs. opposite). WHAM demonstrated the highest accuracy (MAPE:13.61 ± 10.55%) with minimal bias (<10%), consistently performing well across all views and leg sides. OpenPose performed similar to WHAM in the sagittal view (MAPE:14.38 ± 9.63%) but poorly in the frontal view (MAPE:71.33 ± 17.24%) due to substantial underestimation (bias:-71.33 ± 17.24%). VideoPose3D showed poor accuracy across all conditions (MAPE:39.09 ± 20.54%). WHAM also characterized differences in knee flexion kinematics between fall strategies (e.g., least vs. most knee flexion) but did not fully reproduce side-specific kinematic differences between legs, particularly for tuck-and-roll falls. This is the first study to validate pose estimation algorithms for estimating knee impact angles from video-captured falls in older adults. Future work should fine-tune WHAM using fall-specific data to further improve its performance in tracking body movements during falls.

## Introduction

Falls are a leading cause of injury and death in older adults, accounting for 90% of hip fractures, 91% of wrist fractures, and 80% of traumatic brain injury hospitalizations [1,2]. Teaching protective movements, such as bending the knees during a fall, has been shown to reduce injury severity to the hip [3], wrist [4], and head [5]. Knee flexion, when combined with an eccentric braking torque, reduces the velocity of the body at impact [6]. Increased knee flexion during the fall also helps absorb energy and reduce impact forces on the hip [6,7]. The flexed knee posture alters the ground reaction force vector (i.e., closer line of action relative to the hip), producing a smaller moment arm that blunts the external hip torque at impact [8] and lowers the risk of fracture from a fall [9].

Accordingly, quantifying the knee flexion angle at impact provides a key opportunity to evaluate the efficacy of protective fall strategies with respect to injury risk. Despite its significance, the kinematics of knee flexion during falls have not been thoroughly explored due to safety concerns associated with lab-based fall experiments [3] and technical limitations, such as marker occlusions in motion capture systems [10]. As a result, a wide variety of real-life falls—typically accidental and highly variable—cannot be reproduced in laboratory settings. To address these challenges, some studies have analyzed falls captured in security footage from long-term care settings [9,11]; however, these studies were limited to binary observational analyses (e.g., whether the knees were bent) and could not quantify detailed knee joint kinematics.

Recent advances in Artificial Intelligence (AI)-based pose estimation models offer a promising solution to this limitation by enabling efficient, automated extraction of movement kinematics from a video [12]. Previous literature has demonstrated that pose estimation can accurately track the knee joint movement across a range of movement patterns, including gait [13,14], lifting [15], and jumping tasks [16]. Additionally, our previous work showed that a pose estimation algorithm could accurately estimate hip impact velocity during video-captured falls, with a 7.28% error rate [17]; however, no prior studies have examined the accuracy of pose estimation algorithms for estimating joint kinematics during a fall. Estimating joint angles is more challenging, as it requires detecting multiple key points and accurately reconstructing depth and segment alignment, unlike linear measures such as hip impact velocity [12,17].

We aimed to evaluate and compare pose estimation models for analyzing knee kinematics in video-captured falls. Three models were selected based on their relevance to falling movements: *OpenPose*, *VideoPose3D*, and *World-grounded Humans with Accurate Motion* (*WHAM*). OpenPose, a widely used 2D pose estimation tool [12], performs well in sagittal-plane knee kinematics but struggles with frontal views where depth information is critical [18]. To address this, we included two 3D models—VideoPose3D and WHAM—which reconstruct 3D poses from 2D key points [19,20]. VideoPose3D is a fully convolutional deep learning model [19] and is validated for stationary tasks, whereas WHAM, a hybrid deep learning model (i.e., recurrent and convolutional networks), shows promise in accurately tracking knee kinematics across diverse viewing angles [20]. We hypothesized that WHAM would yield superior accuracy (i.e., smaller mean absolute error) compared to the other two models due to its advanced capacity for 3D kinematic tracking. Accordingly, this work is framed as a controlled laboratory validation of pose estimation models and represents an essential first step toward assessing their potential applicability to real-world falls.

## Materials and methods

### Overview of study design

This study evaluated the accuracy of three pose estimation models—OpenPose, VideoPose3D, and WHAM—for estimating knee flexion angles at ground impact during video-captured falls. A total of 121 fall trials from 13 older adults were analyzed. For each trial, video recordings were processed using each pose estimation model to generate knee joint angle time series. Knee impact angles were extracted and compared with ground truth measurements obtained simultaneously using a motion capture system. Agreement between pose-estimated and motion capture–derived knee angles was quantified using mean absolute error (MAE), mean absolute percentage error (MAPE), and bias. Analyses were performed across different viewing planes (sagittal vs. frontal) and leg sides (impacted vs. opposite). In addition, the discriminative ability of the best-performing model to detect differences in knee kinematics among fall strategies (knee block, stick-like, and tuck-and-roll) was evaluated.

### Participants

This is a secondary analysis of the randomized controlled trial of a safe landing techniques training study (NCT03540082) [10]. All procedures were approved by the University of Illinois at Urbana-Champaign Institutional Review Board and experiments followed relevant guidelines and regulations. Prior to participation in the study, all participants provided written informed consent. Data collection was conducted between July 25, 2017, and October 11, 2017. The data were accessed for the purpose of this study on May 10, 2023. To be included, participants had to be healthy individuals capable of safely performing the study procedures, characterized by the following: 1) aged between 55−75 years, 2) weight between 45−100 kg, 3) height between 150−195 cm, 4) bone mass density t-score > −1.0, 5) sit-to-stand test completed 5 times within 10 seconds, 6) Montreal Cognitive Assessment (MoCA) score > 26. Participants were excluded if they had any of the following conditions: 1) bone fracture in the past 5 years, 2) diagnosis of osteoporosis or osteopenia, 3) history of neuromuscular disease or stroke, 4) difficulty rising from a prone position, 5) pregnancy, and 6) a tendency to easily bruise or delicate skin. These inclusion and exclusion criteria yielded a sample of physically healthy older adults.

### Falling experimental setup

Participants fell sideways onto a crash pad by releasing a tether attached at a 10° lean [10], with randomized delays (3–8 s) to introduce unpredictability [10]. They were instructed to land naturally without arm extension or stepping to isolate lower limb impacts. Trials were randomized between right- and left-side falls [10].

### Video recording setup

A digital RGB video camera recorded the participant from the frontal view at 30 frames per second (1920 × 1080 resolution). The camera was positioned 110 cm above the ground, level with the participant, who stood 45 cm above the floor due to the platform and crash pad. Videos were cropped to show only the participant and trimmed to capture the fall—from tether release to ground contact (mean duration: 2.42 seconds).

Classification of videos – fall strategies, leg side, and plane of view

**Fig 1:**
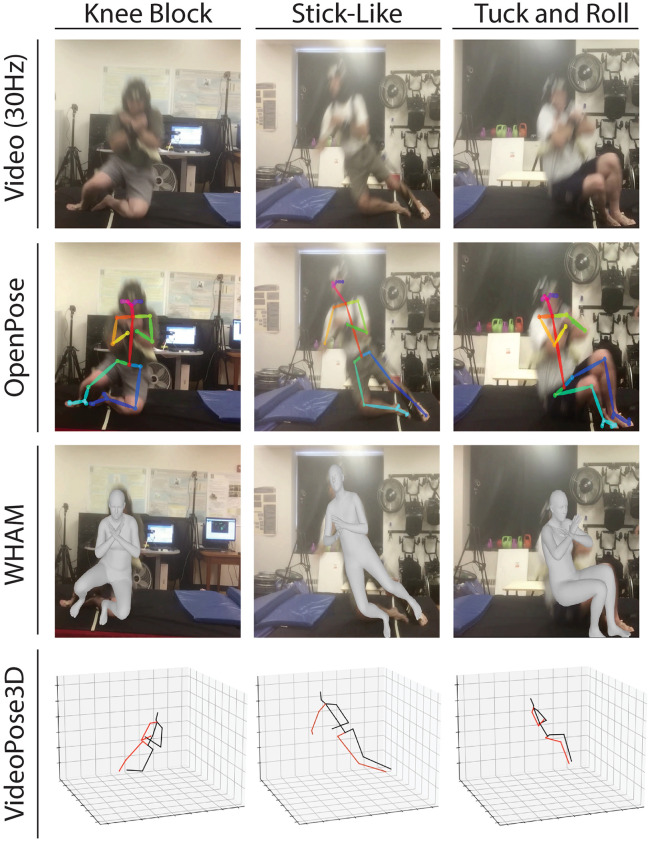
Example video frames showing human participant falls, overlaid with key point detection from OpenPose and 3D pose reconstruction from VideoPose3D and WHAM. Participants utilized diverse movement strategies during the fall, such as blocking the fall with their knee (referred to as ‘knee block’), falling rigidly (described as a ‘stick-like fall’), or employing a tuck-and-roll strategy.

Participants exhibited three fall strategies: knee block, stick-like, and tuck-and-roll (Fig 1). “Knee block” involved knee contact preceding thigh or hip contact. “Stick-like” falls involved maintaining initial sideways orientation and first contacting the lateral leg or hip. In the original study, some trials required participants to utilize a “tuck-and-roll” strategy, where they bent their knees while falling, rotated their bodies backward, landed on their posterior hips, and rolled along their backs [10]. Leg sides were classified as “impacted” (same side as fall direction) or “opposite” (opposite side) (Fig 1).

Lastly, we classified each video based on the predominant plane of the view of the knee for each leg side – either the frontal or sagittal plane. This classification was important as knee flexion-extension accuracy depends on the viewing plane. Sagittal views allow optimal capture of knee motion, while other planes are suboptimal (Fig 1), which can significantly affect knee angle estimation accuracy in video analysis [18].

### Data collection and analyses for ground truth measurement

To validate the abilities of the three pose estimation tools in accurately estimating knee joint angle at impact, a motion capture system served as the ground truth.

Kinematic data of the hip, thigh, knee, shank and ankle were collected bilaterally using motion capture cameras (Vicon, Oxford Metrics, Oxford, England) at 100 Hz. The 3D positions were digitally filtered using a fourth-order, zero-phase, low-pass Butterworth filter with a cutoff frequency of 10 Hz [21]. Knee angles were calculated for each frame using the inverse cosine of the angle between the hip-knee and knee-ankle segments (Eq 1). Finally, the knee impact angles for each leg side were calculated as the knee angle at the time of impact (t). Time of impact was defined as the frame where the vertical trajectory (Z) of the impacted body part reached zero, corresponding to each of the three fall strategies (knee block: knee; stick-like: anterior hip; tuck and roll: posterior hip).


$$\begin{array}{c} \theta_{knee}(t)=\;{cos}^{-\text{1}}\frac{{\left(r_{hip}(t)-r_{knee}(t)\right)}^{T}\left(r_{ankle}(t)-r_{knee}(t)\right)}{∥r_{hip}(t)-r_{knee}(t)∥∥r_{ankle}(t)-r_{knee}(t)∥} \end{array}$$ (Eq.1)

### Pose estimation video analysis

**Fig 2:**
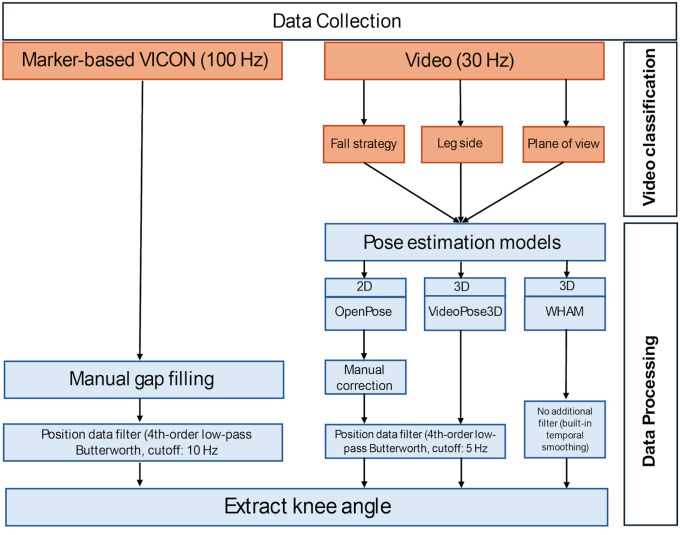
Flowchart of classification and data analysis processes for motion capture and video data. For OpenPose, trajectories of the position data at the hip, knee, and ankle key points (i.e., joint centers) were visually inspected to correct for erroneous switching between left and right limbs and misidentified body parts for a given frame, and the key point was deleted for these frames. Specifically, across the 121 video files, a total of 11,496 data points (i.e., 5,748 frames per side for each joint) were analyzed for each joint across all video recordings. Of these, 100 frames (3% of total frames) required correction of hip key points, 153 frames (5%) required correction of knee key points, and 241 frames (8%) required correction of ankle key points. Because OpenPose outputs were manually inspected and corrected for left-right limb switching and body-part misidentification prior to analysis, the OpenPose pipeline evaluated in this study represents a curated, semi-automated workflow rather than a fully automated pose estimation workflow. These corrections were performed blinded to the ground truth. We filled gaps in key point position trajectories using linear interpolation for gaps spanning up to four video frames. We then filtered the position data using a zero-lag 4th order low-pass Butterworth filter with a cutoff frequency at 5 Hz, following the previous methods that used OpenPose [17,23].

Video recordings were processed using three different pose estimation models: OpenPose [22], VideoPose3D [19], and WHAM [20] (Fig 2).

For 3D pose estimation (i.e., VideoPose3D and WHAM), both models detect key points by frame and construct 3D global joint coordinates that align with the original video pixels [19,20]. Unlike the OpenPose analysis, the 3D coordinates generated from these 3D pose estimation models could not be manually corrected due to the inability to accurately determine 3D positions from a 2D video using human vision. Therefore, these algorithms were evaluated using their native automated processing pipelines without comparable manual intervention. For VideoPose3D, we filtered the position data of the hip, knee, and ankle using a zero-lag 4th order low-pass Butterworth filter with a cutoff frequency at 5 Hz. We did not apply an additional filter for WHAM, since the algorithm itself incorporates temporal encoders that inherently smooth joint trajectories [20].

**Fig 3:**
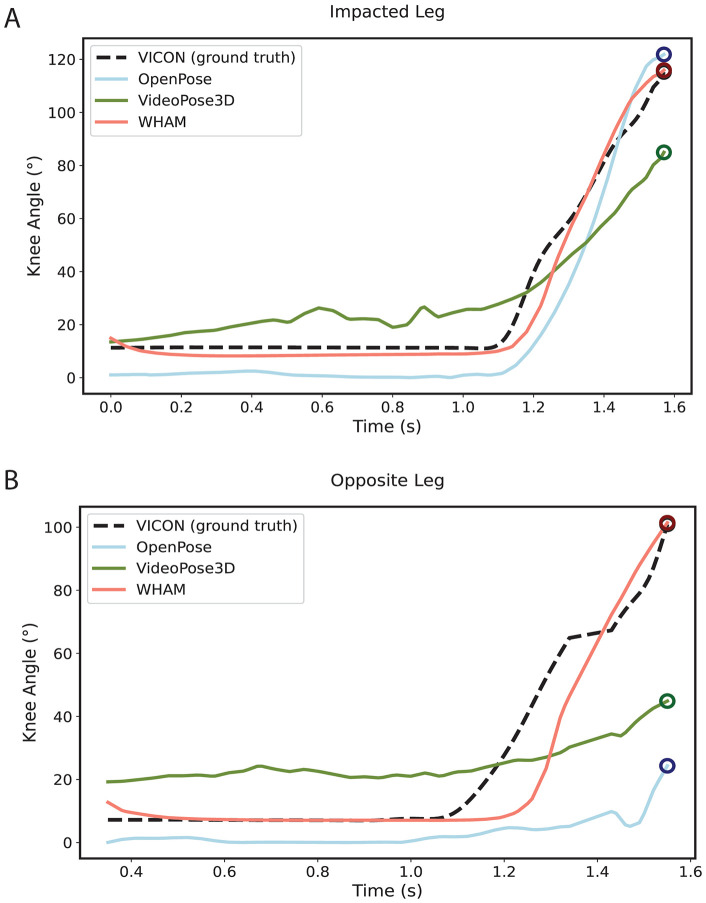
Time profile of knee flexion angle for a representative participant in a single sideways fall trial, captured concurrently using motion capture (VICON), and a video analyzed by OpenPose, VideoPose3D, and WHAM. (A) Impacted leg captured in the sagittal plane. (B) Opposite-side captured in the frontal plane. Colored circles indicate the knee impact angle for each method of measurement.

For OpenPose and VideoPose3D, knee angles were calculated from joint positions using the vector operation (Eq 1), while WHAM directly provided knee joint angles [20]. Finally, impact angles for each leg were extracted at the frame where ground impact occurred—that is, the first frame where any lower body part (i.e., knee or hip) contacted the ground — identified through visual inspection (Fig 3). Impact identification was performed independently by two raters (RM and YM), with any disagreements resolved through discussion. Agreement between raters was high. Of the 121 video files, 68 (56.2%) showed exact agreement in impact frame identification, 43 (35.5%) differed by 1 frame, and 10 (8.3%) differed by 2 frames. No cases exceeded a two-frame discrepancy. To further evaluate the potential impact of this discrepancy, we conducted an additional analysis examining frame-to-frame changes in knee angle (30 Hz). Based on VICON data (gold standard), the mean change in knee angle over one frame (0.033 s) was 4.58 ± 2.76° for the impact side and 2.68 ± 2.18° for the opposite side. Relative to the average knee impact angles (98.88° for the impact side and 89.71° for the opposite side), these differences correspond to approximately 4.6% and 3.0%, respectively, indicating minimal influence of ±1 frame uncertainty.

### Statistical analyses

All statistical analyses were performed in R (version 4.5.1, R Foundation for Statistical Computing, Vienna, Austria) using the *lme4*, *lmerTest*, and *emmeans* packages. All analyses used two-sided tests with statistical significance set at α = 0.05.

**Validation metrics:** We calculated mean absolute error (MAE=|Pose tool−Ground truth|), and mean absolute percentage error ($MAPE=\frac{\left|Pose\;tool-Ground\;truth\right|}{Ground\;truth}\times \text{1}\text{0}\text{0}\%)$. Additionally, we identified biases (=Pose tool−Ground truth) and percentage bias $\left(=\frac{Pose\;tool-Ground\;truth}{Ground\;truth}\times \text{1}\text{0}\text{0}\%\right)$, which pertain to the scale and orientation (i.e., over or underestimations) of the errors. Bland-Altman plots were also generated to visually assess bias of each pose estimation tool. Bland-Altman limits of agreement were calculated using the standard approach [24] and do not account for the repeated-measures structure of the data. Accordingly, the limits of agreement should be interpreted as descriptive rather than inferential estimates.

**Comparison of validation metrics among pose tools:** Differences in MAE and bias across pose estimation tools and viewing planes were assessed using linear mixed effects models. Pose estimation tool, viewing plane, and their interaction were modeled as fixed effects. A random intercept for participant ID and a nested random intercept for trial within participant were included to account for within-subject correlation across repeated measurements and within-trial correlation across pose estimation tools and leg sides. Type III analysis of variance with Satterthwaite’s method was used to evaluate fixed effects. When a significant interaction effect was observed, post hoc pairwise comparisons were performed using estimated marginal means with Sidak adjustment for multiple comparisons.

**Discriminative validity of pose tools:** Then, to assess the ability of the best-performing pose estimation model to detect differences in knee impact angles across fall strategies and leg sides, separate linear mixed effects models were constructed for ground truth (VICON) and pose-estimated measurements. Fall strategy, leg side, and their interaction were specified as fixed effects. A random intercept for participant ID and a nested random intercept for trial within participant were included to account for within-subject correlation across repeated measurements and within-trial correlation across leg sides. Fixed effects were evaluated using type III analysis of variance with Satterthwaite’s method. When appropriate, post hoc comparisons between leg sides were conducted within fall strategies using estimated marginal means with Sidak adjustment.

## Results

### Participant characteristics

A total of 13 participants underwent the study procedures (Table 1). The participants’ ages ranged from 55 to 73 years, with an average of 64.0 ± 5.9 years, and ten of the 13 participants (77%) were male. Further, all participants were physically and cognitively healthy, as indicated by their normal Bone Mass Density t-scores, 5 times sit to stand times, and high MoCA scores.

**Table 1:** Participants demographics.

Characteristic	Mean ± SD
N	13 (3F/10M)
Age (years)	64.0 ± 5.9
Height (cm)	171.1 ± 7.9
Weight (kg)	73.6 ± 13.0
Bone Mass Density (t-score)	0.17 ± 0.96
5 times sit to stand (sec)	7.60 ± 1.2
MoCA	1.1 ± 1.3

### Characteristics of classification of videos – fall strategies, leg side, and plane of view

A total of 121 falls were analyzed: 58 knee block (48%), 30 stick-like (25%), and 33 tuck-and-roll (27%). To assess potential sources of pose estimation error, we classified the viewing plane of each knee near impact. For the impacted-side leg, most videos (109; 90%) captured the sagittal plane. For the opposite-side leg, the frontal plane predominated (78; 64%) (Table 2). Knee block and stick-like falls were generally recorded with the impacted-side leg in sagittal and the opposite leg in frontal planes, while tuck-and-roll falls were typically captured in the sagittal plane for both legs (Table 2).

**Table 2:** Knee configuration characteristics of fall strategies. Knee block and stick-like falls were mainly captured with the impacted-side leg in the sagittal plane and the opposite-side leg in the frontal plane, whereas tuck-and-roll falls were predominantly recorded in the sagittal plane for both legs.

	Impacted-side Leg		Opposite-side Leg	
	**Sagittal N (%)**	**Frontal N (%)**	**Sagittal N (%)**	**Frontal N (%)**
**Total (N = 121)**	**109 (90%)**	**12 (10%)**	**43 (36%)**	**78 (64%)**
Knee block (N = 58)	57 (98%)	1 (2%)	6 (10%)	52 (90%)
Stick-like (N = 30)	20 (67%)	10 (33%)	5 (17%)	25 (83%)
Tuck-and-roll (N = 33)	32 (97%)	1 (3%)	32 (97%)	1 (3%)

### Characteristics of knee impact angle

The knee impact angle measured using the ground truth method (i.e., VICON motion capture) showed substantial variability in knee impact angles by leg side and fall strategy (Table 3). Linear mixed effects modeling revealed significant main effects of leg side (F(1, 118) = 32.68, p < 0.01) and fall strategy (F(2, 50.38) = 25.19, p < 0.01), and a significant interaction between fall strategy and leg side (F(2, 118.00) = 3.74, p = 0.027), indicating that the difference in knee flexion between impact and opposite legs varied by fall strategy. Post hoc comparisons within each fall strategy revealed greater knee flexion on the impacted vs. opposite leg for both stick-like and knee block fall strategies (all p-values < 0.01), but no difference in knee flexion between leg sides for the tuck-and-roll strategy (p = 0.33).

**Table 3:** Characteristics of knee impact angle (°) recorded by motion capture. Stick-like falls showed the least knee flexion on both legs, knee block falls demonstrated greater flexion on the impacted side, and tuck-and-roll falls exhibited the greatest bilateral knee flexion.

Leg Side	Fall Strategy	Mean	S.D.	S.E.	Min	Max
**Impacted**	**All (N = 121)**	**98.88**	**17.79**	**1.62**	**51.02**	**135.36**
	Knee block	103.98	14.67	1.92	68.61	133.98
	Stick-like	82.23	15.67	2.86	51.02	109.23
	Tuck-and-roll	105.03	15.36	2.67	72.16	135.36
**Opposite**	**All (N = 121)**	**89.71**	**21.37**	**1.94**	**36.26**	**134.04**
	Knee block	92.51	16.88	2.21	52.12	134.04
	Stick-like	70.39	23.13	4.22	36.26	104.24
	Tuck-and-roll	102.35	13.85	2.41	68.13	127.68

Kinematic patterns observed across strategies also aligned with their original descriptions: stick-like falls produced the lowest amount of bilateral knee flexion (e.g., “fall like a stick”), while tuck-and-roll falls exhibited the greatest bilateral flexion compared to the other strategies consistent with the instruction to “bend your knees” [10]. Knee block falls demonstrated an asymmetric pattern, with increased knee flexion on the impacted leg and relatively lower flexion on the opposite leg.

### Descriptive characteristics of validation metrics for the three pose estimation algorithms

The validation metrics for knee angle estimation using the three pose estimation algorithms varied substantially depending on the viewing plane and leg side (Table 4). Notably, the MAE and MAPE results show that WHAM was the most robust tool, demonstrating the highest accuracy among the pose estimation algorithms across diverse video recording conditions involving different viewing planes and leg sides (all MAPEs < 15%, see Table 4). Specifically, in the sagittal view (i.e., optimal view for measuring knee flexion angle), OpenPose (MAPE: 14.37 ± 9.62%) and WHAM (MAPE: 12.83 ± 10.77%) yielded similar accuracy for both leg sides. However, in the frontal view, which is a suboptimal view for measuring knee angle, only WHAM maintained a high level of accuracy for both leg sides (MAPE: 14.93 ± 10.03%) comparable to that of the sagittal view. In contrast, OpenPose’s performance became markedly poorer (MAPE: 71.33 ± 17.24%) compared to the sagittal view. VideoPose3D consistently showed poor performance irrespective of viewing plane or leg side (MAPE: 39.09 ± 20.54%).

**Table 4:** Validation metrics (MAE, MAPE, bias, and percent bias) for knee angle, analyzed by viewing plane, leg side, and pose estimation tool. Estimation accuracy varied by viewing plane and leg side, with WHAM consistently showing the highest accuracy (i.e., lowest error) across all conditions, while OpenPose performed poorly in the frontal view and VideoPose3D showed poor accuracy across all conditions.

Sagittal view (N = 152)					
Leg Side	Pose Tool	Mean Absolute Error (°)	Mean Absolute Percentage Error (%)	Bias (°)	Percent Bias (%)
Impacted (N = 109)	OpenPose	14.40 ± 9.99	14.22 ± 9.34	−9.41 ± 14.83	−8.8 ± 14.61
	VideoPose3D	39.06 ± 20.67	38.80 ± 20.05	−37.62 ± 23.21	−37.04 ± 23.18
	WHAM	12.46 ± 9.50	13.36 ± 11.60	7.29 ± 13.91	8.86 ± 15.34
Opposite (N = 43)	OpenPose	14.23 ± 9.46	14.76 ± 10.43	−10.76 ± 13.35	−10.58 ± 14.73
	VideoPose3D	30.41 ± 19.62	30.07 ± 19.32	−28.64 ± 22.17	−28.23 ± 21.99
	WHAM	11.26 ± 7.92	11.56 ± 8.41	5.67 ± 12.63	6.53 ± 12.80
**Frontal view (N = 90)**					
**Leg Side**	**Pose Tool**	**Mean Absolute Error (°)**	**Mean Absolute Percentage Error (%)**	**Bias (°)**	**Percent Bias (%)**
Impacted (N = 12)	OpenPose	52.35 ± 29.05	58.99 ± 27.54	−52.35 ± 29.05	−58.99 ± 27.55
	VideoPose3D	32.80 ± 22.79	36.61 ± 22.78	−28.54 ± 28.36	−29.80 ± 31.82
	WHAM	12.95 ± 9.76	14.43 ± 10.32	4.63 ± 15.96	5.25 ± 17.43
Opposite (N = 78)	OpenPose	62.12 ± 22.03	73.23 ± 14.41	−62.12 ± 22.03	−73.23 ± 14.41
	VideoPose3D	39.28 ± 20.12	44.84 ± 19.98	−36.34 ± 25.10	−39.53 ± 29.21
	WHAM	12.21 ± 7.74	14.96 ± 10.05	−6.35 ± 13.04	−7.09 ± 16.63

Bias analysis further revealed that the inaccuracy of OpenPose in the frontal view was due to substantially underestimated knee flexion angles (−71.33 ± 17.24%), while it maintained minimal bias in the sagittal view (< 10% underestimation). In contrast, WHAM showed only negligible overestimation (< 10%) across all viewing planes and leg side conditions. Additionally, bias analysis showed that the inaccuracy of the VideoPose3D was driven by underestimation of the knee flexion angles (−36.95 ± 25.45%).

### Statistical comparison of the validation metrics for the three pose estimation algorithms and planes of view

**Fig 4:**
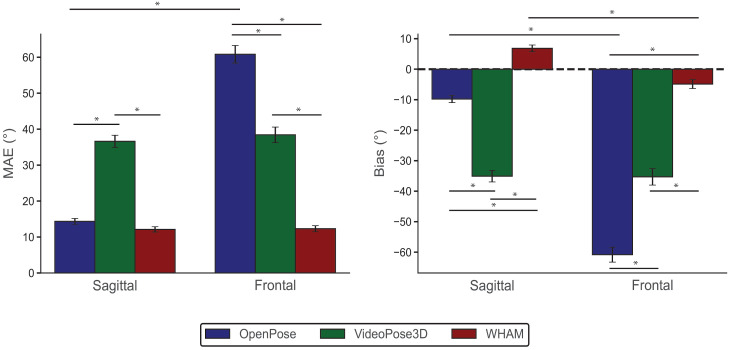
Group comparisons of OpenPose, VideoPose3D, and WHAM agreement with the ground truth. Bar plots showing performance differences between pose tools across different view planes (sagittal, frontal; x-axis), assessed by (A) the mean MAE, and (B) the mean bias (y-axis). Error bars represent standard deviation. *p < 0.01.

We next examined differences in MAE and bias across pose estimation tools and viewing planes using linear mixed effects models. For MAE, significant main effects were observed for pose estimation tool (F(2,596.32) = 144.96, p < 0.01), plane of view (F(1,719.63) = 200.57, p < 0.01), and their interaction (F(2, 596.32) = 182.89, p < 0.01). Post-hoc comparisons showed significantly lower MAEs for WHAM and OpenPose versus VideoPose3D in the sagittal view (p < 0.01; Fig 4A), and significantly lower MAEs for WHAM versus both OpenPose and VideoPose3D in the frontal view (p < 0.01). Only OpenPose demonstrated significant differences in MAE between sagittal and frontal views (p < 0.01).

Bias analysis also revealed significant main effects of pose estimation tool (F(2,598.76) = 144.20, p < 0.01), plane of view (F(1,699.99) = 243.64, p < 0.01), and their interaction (F(2,598.76) = 141.87, p < 0.01). Post-hoc analyses indicated that VideoPose3D underestimated knee angles relative to WHAM in both planes (p < 0.01; Fig 4B), and OpenPose underestimated relative to WHAM in the frontal view (p < 0.01). In the sagittal view, OpenPose slightly underestimated (−9.79 ± 14.40°) while WHAM slightly overestimated (6.83 ± 13.54°) the knee impact angle (Fig 4B).

### Systematic bias analysis through Bland-Altman plots

**Fig 5:**
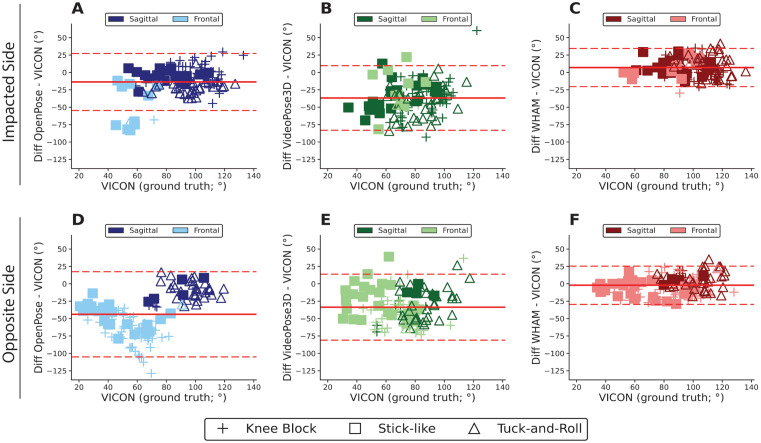
Bland-Altman plots of the knee impact angle for OP, VP3D, and WHAM compared with that of the ground truth (VICON). (A) OpenPose, (B) VideoPose3D, and (C) WHAM show the knee impact angle of the impacted side leg, and (D), (E), and (F) show the knee impact angle of the opposite side leg. Limits of agreement are specified as the average difference (red solid line) and 95% confidence interval of the error (red dotted line). Videos are grouped by (i) the fall strategy utilized during the fall and (ii) the plane of view of the video.

WHAM showed the narrowest limits of agreement (LoA) for both the impacted (−20.44° to 34.50°; Fig 5C) and opposite legs (−29.57° to 25.42°; Fig 5F), indicating highest agreement with ground truth. In comparison, OpenPose showed moderately wide LoA for the impacted leg (−54.68° to 27.34°; Fig 5A) and substantially wider LoA for the opposite leg (−105.07° to 17.33°), driven by significant underestimations of knee flexion on the opposite leg in frontal views (Fig 5D, light blue squares). VideoPose3D showed no consistent bias but greater error dispersion across all conditions (LoA impacted: −83.17° to 9.73°; LoA opposite: −81.03 to 13.81°; Fig 5B and E). WHAM maintained narrow LoA and consistent spread across both planes and leg sides (Fig 5C and F).

### Discriminative validity of WHAM

**Fig 6:**
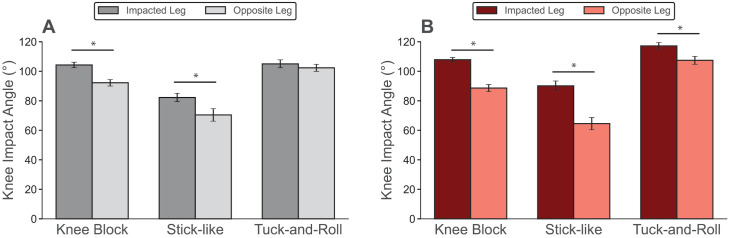
Comparative analysis of knee impact angle across leg sides, separated by fall strategy. (A) Grouped bar plot of the knee impact angle measured by the ground truth (VICON), grouped by fall strategy and further separated by leg side. (B) Grouped bar plot of the knee impact angle measured by WHAM. Error bars represent standard deviation; *p < 0.01.

We next determined whether WHAM, which showed the best accuracy among the pose estimation tools, could accurately distinguish variations in the knee impact angle across fall strategies and leg sides (Fig 6). We evaluated the correspondence of WHAM estimates with the ground truth findings (see section Characteristics of knee impact angle).

Knee impact angles estimated by WHAM also varied by fall strategy and leg side. Linear mixed modeling revealed significant main effects of leg side (F(1, 118) = 174.79, p < 0.01) and fall strategy (F(2, 75.73) = 43.38, p < 0.01), as well as a significant interaction between leg side and fall strategy (F(2, 118) = 9.62, p < 0.01). These patterns were consistent with the ground truth measurements

Post hoc comparisons within each fall strategy showed that WHAM accurately characterized the between-leg knee flexion trends observed in the ground truth for stick-like and knee-block strategies (Fig 6). WHAM also captured the general between-strategy differences in knee flexion seen in the ground truth, where stick-like falls produced the lowest bilateral knee flexion, knee block falls showed increased knee flexion on the impacted leg and relatively lower flexion on the opposite leg, and tuck-and-roll falls yielded the greatest bilateral knee impact angle (Fig 6A and B). However, WHAM did not replicate the within-strategy kinematic pattern of tuck-and-roll falls, where there was virtually no difference in knee impact angle between legs in the ground truth (mean diff = 2.68°; Fig 6A). In contrast, WHAM overestimated knee flexion in the impact leg relative to the opposite leg (p < 0.01, mean diff = 9.74°; Fig 6B), highlighting an area for model improvement.

## Discussion

The purpose of this study was to assess the validity of three pose estimation models—OpenPose, VideoPose3D, and WHAM—in estimating knee flexion angle at ground contact during a fall (i.e., knee impact angle) using video-captured falls in older adults (N = 13, age: 64.0 ± 5.9 years). WHAM showed the highest accuracy (MAPE: 13.61 ± 10.55%), achieving the lowest error among the pose estimation tools across all viewing planes and leg sides. OpenPose achieved a similar level of error to WHAM only in the sagittal view (MAPE: 14.38 ± 9.63%), but showed substantially poorer performance in the frontal view (MAPE: 71.33 ± 17.24%). VideoPose3D consistently demonstrated poor accuracy across all conditions (MAPE: 39.09 ± 20.54%). However, while WHAM captured differences in knee flexion kinematics between fall strategies (e.g., least vs. most knee flexion), it did not fully reproduce side-specific kinematic differences between legs, particularly for tuck-and-roll falls, where it overestimated the knee angle on the impact side.

Our primary finding was that WHAM achieved the lowest error in estimating knee impact angle among the pose estimation tools across the plane of views (sagittal MAE: 12.12 ± 9.07°, frontal MAE: 12.31 ± 7.98°), with minimal bias across leg sides (impacted: + 7.02 ± 14.08°, non-impacted: −2.07 ± 14.08°). These findings align with Shin et al., who reported that WHAM accurately tracked knee flexion time series during stair ascent [20]. Despite higher fall velocities (2.32 m/s vs. 0.40–0.60 m/s in stair ascent) [17,25], WHAM yielded modest error magnitudes (13.61% MAPE) even under rapid movements and blurred images, demonstrating its robustness for 3D kinematics in fall-related applications.

Although WHAM demonstrated the least error (MAE: 12.23 ± 8.80°) among the pose estimation algorithms, the extent to which this level of error affects clinical interpretation (e.g., fracture risk) remains unclear [6]. Previous work has linked greater knee flexion during falls to reduced impact severity; however, these findings were derived from comparisons between movement strategies (e.g., squat vs. non-squat) without directly quantifying knee flexion angle at impact. As a result, clinically meaningful thresholds of knee flexion that distinguish injurious from non-injurious falls remain undefined. Future work should aim to use pose-estimated knee angles to help establish these thresholds, providing a basis for determining the level of measurement accuracy required for clinically meaningful interpretation [26].

Additionally, WHAM’s ~12° error in knee flexion estimates may limit its ability to capture side-specific differences between legs for tuck-and-roll strategy. For tuck-and-roll falls, knee flexion was similar between legs in the ground truth (mean difference = 2.68°), whereas WHAM estimated substantially greater flexion on the impact leg relative to the opposite leg (mean difference = 9.74°). Because WHAM’s average error (~12°) exceeds this mean difference between the legs, the model may overestimate the knee angle on the impact side and produce statistically significant differences where none exist. This illustrates that relatively lower absolute error does not guarantee valid discrimination of small between-condition differences. Nevertheless, WHAM preserved the overall direction of between-strategy differences (i.e., discriminative validity), despite error magnitudes that limit precise agreement with the ground truth. Specifically, WHAM was able to capture the ‘flexed’ nature of knee movement on the impact side for both tuck-and-roll and knee block strategies, in contrast to the more ‘extended’ pattern observed in stick-like falls. Future work should focus on improving WHAM’s accuracy in estimating knee flexion in tuck-and-roll falls, potentially by incorporating fall-specific training data [27].

It is also worth noting that WHAM’s observed error may be partly attributable to differences in angle definition between systems. WHAM provides joint angles directly from its internal kinematic model [20], whereas VICON and the other pose estimation tools derived angles from a vector-based hip-knee-ankle calculation. This definitional difference represents a plausible source of systematic bias independent of tracking accuracy, and future work should look to standardize angle extraction approaches across tracking methods.

Our finding that OpenPose performed poorly for frontal plane knee movements aligns with previous research showing large deviations from motion capture during frontal plane knee flexion in a lunge task (MAE: 31.75°), while sagittal plane estimates were more accurate (MAE: 10.57°) [18]. A key limitation of 2D motion analysis methods, including pose estimation, is difficulty capturing out-of-plane movements such as frontal plane knee kinematics during squatting or falling [21,28]. In our falls, projecting 3D knee flexion into 2D likely distorted the kinematics, causing significant underestimation of knee impact angles by OpenPose [12].

VideoPose3D showed poor performance under all conditions (MAPE: 39.09 ± 20.54%). This inaccuracy is likely due to an underlying assumption of the model, which requires a smooth, stable center of mass (COM) trajectory (i.e., two feet on the ground, lifting tasks) to produce accurate kinematics [19,29]. We therefore postulate that the rapid downward shift of the COM during a fall [30] contributes to the model’s inability to accurately track body movement, resulting in large errors in knee impact angle estimation by VideoPose3D.

Previously, we demonstrated that hip impact velocity estimated by OpenPose yielded good accuracy, with a 7.28 percent error [17]. In that study, OpenPose’s 2D analysis provided consistent results across different viewing angles because hip impact velocity was calculated only from the vertical component of the hip position, which does not change with the plane of view [17]. In contrast, the current study highlights that estimating knee angles is more challenging because it involves angular kinematics. Both OpenPose and WHAM produced similar knee angle estimates (< 15% error) in the sagittal view; however, only WHAM’s 3D analysis maintained this level of performance in the frontal view, where accurate depth estimation and the spatial relationship between body segments are more difficult to capture. These performance differences are largely methodological, reflecting fundamental differences in model architecture and training datasets. OpenPose estimates 2D joint locations in the image plane and therefore lacks explicit depth modeling [22], whereas WHAM uses learned kinematic and motion priors from large motion capture datasets (e.g., AMASS) to map temporally consistent 2D key points onto a world-grounded representation, resulting in a full-body reconstruction of the 3D pose [20,31]. These priors encode typical spatial relationships and coordination patterns between body segments, enabling more robust estimations of knee joint geometry during multi-planar movements [20,32]. While WHAM was trained with a richer training dataset and superior methodology for capturing 3D kinematics compared to OpenPose, it is unknown which factor contributes more to WHAM’s superior performance. Nevertheless, these findings emphasize the importance of selecting the appropriate pose estimation algorithm based on the specific biomechanical parameter and video recording conditions.

While our study focused on single-camera 3D pose estimation for falls, other systems exist that achieve high accuracy for lower-limb joint kinematics in controlled laboratory tasks. For example, alternative approaches like markerless motion capture (e.g., Theia3D) and multi-camera pose estimation pipelines (e.g., OpenCap) have demonstrated low errors for estimating knee angles during gait (RMSE: 3.3°; [33]) and sport movements (MAE: 4.58–10.91°; [34]). However, these approaches require controlled environments, synchronized camera systems, or specialized calibration procedures [33,35]. Although they achieve lower joint angle errors, such requirements limit their applicability for fall analysis in real-world settings, where falls are unexpected, occur in unconstrained environments, and are rarely captured with optimal camera placement or multi-view coverage [9]. In contrast, a single camera pose estimation framework is more compatible with practical real-world constraints, such as surveillance cameras or in-home monitoring systems. Our findings indicate that WHAM offers a favorable balance between accuracy and potential ecological validity for estimating knee impact angles from monocular video. In particular, WHAM appeared less sensitive to viewing-plane effects than OpenPose, whose accuracy declined markedly in the frontal view, whereas VideoPose3D performed poorly regardless of viewing plane. However, the distribution of viewing planes was inherently confounded with both leg side and fall strategy due to the naturalistic constraints of the fall videos (Table 2). Consequently, we were unable to fully disentangle the independent effects of viewing plane, leg side, and fall strategy on pose-estimation error. These findings should therefore be interpreted with appropriate caution.

This study has several limitations. First, it should be noted that the WHAM algorithm was trained on diverse datasets of intentional movements [31] and not accidental movements like falls. Potentially, WHAM’s ability to track knee impact angles for different body configurations could be improved by fine-tuning the algorithm [29] on a dataset that contains video-recorded falls. Fall trials were also recorded under controlled conditions (e.g., stable lighting, fixed camera placement, no background clutter), and accordingly, our findings reflect pose estimation performance in an optimal recording setup. Future studies should explore the effects of variable camera positioning, video resolution, background clutter, and segmental occlusions on pose estimated knee angles to better approximate real-world fall conditions [32,36]. In addition, preprocessing differed across the pose estimation algorithms, which may have affected their relative performance. OpenPose outputs underwent manual inspection and correction of clearly erroneous key-point assignments, whereas VideoPose3D and WHAM were evaluated using fully automated workflows without comparable manual intervention. Consequently, the reported OpenPose performance reflects a curated, semi-automated implementation rather than a fully automated pipeline. Although the proportion of corrected frames was small, these preprocessing differences should be considered when interpreting comparisons among methods and when applying these tools in practice.

Another important limitation is the lack of synchronization between the VICON and video data streams. This dataset is a secondary analysis of a study in which video recordings were collected to supplement VICON-based movement analysis rather than to validate pose-estimation algorithms. As a result, synchronization across measurement systems was not implemented in the original study design. Although impact events were carefully aligned across systems using a standardized procedure with high inter-rater agreement (see the Pose Estimation Video Analysis section), the lack of synchronization remains a potential source of error. Because knee angles can change rapidly during a fall, even small timing discrepancies may have influenced the estimated knee flexion error. Future studies should digitally synchronize these measurement systems to improve the accuracy of pose-estimated kinematic measures. Our experiments also used a tether-release and crash pad method to induce falls in a laboratory environment [10], which does not reflect the characteristics of real-life falls (i.e., accidental, rigid flooring). Additionally, we only addressed the leg configuration at impact (i.e., knee impact angle) for sideways falls, but there are several important body configurations that should be assessed with both sideways falls (i.e., trunk flexion angle [3], hip external rotation [37]) and forward falls (upper body protective responses [38]). Finally, although the total number of analyzed falls was relatively large (N = 121), all data were obtained from only 13 physically healthy older adults performing induced falls in a laboratory setting. This limits the representativeness of the dataset and constrains inference at the population level. While the repeated-measures structure improves internal consistency and supports model comparison, it does not substitute for participant diversity. Accordingly, the findings of this study should be interpreted as a controlled laboratory validation rather than evidence of direct applicability to real-world falls.

## Conclusion

This study presents the first systematic validation of pose estimation algorithms for estimating knee impact angles from video-captured falls in older adults. By comparing OpenPose, VideoPose3D, and WHAM against motion capture ground truth, we show that accurate knee kinematics estimation during falls is achievable but strongly dependent on algorithm choice and viewing conditions. WHAM demonstrated the highest accuracy and minimal bias among the pose estimation algorithms across fall strategies, leg sides, and viewing planes, including frontal views where OpenPose substantially underestimated knee flexion and VideoPose3D performed poorly. WHAM was able to capture overall differences in knee flexion kinematics across fall strategies (e.g., least vs. most knee flexion), but did not fully reproduce side-specific differences between legs, particularly in tuck-and-roll falls. Taken together, these findings establish WHAM as the most consistent and accurate approach among the evaluated models for analyzing fall biomechanics within controlled laboratory settings, while still highlighting important limitations in capturing finer kinematic distinctions. While the present results should not be interpreted as evidence of direct applicability to real-world falls, they provide a necessary validation foundation for future work aimed at improving model performance through fall-specific training data and extending evaluation to more diverse populations and ecologically realistic fall environments.

## Supporting information

S1 File Trial-level data set and R analysis code. (ZIP)
